# Bioactive compounds and functional properties of Rambai (*Baccaurea motleyana* Müll. Arg.) fruit: A comprehensive review

**DOI:** 10.1002/fsn3.2661

**Published:** 2021-11-23

**Authors:** Pradip Debnath, Sayed Koushik Ahmad, Rezwan Ahmed Mahedi, Amlan Ganguly, Kishore Kumar Sarker

**Affiliations:** ^1^ Department of Pharmaceutical Technology Jadavpur University Kolkata India; ^2^ Department of Pharmacy Comilla University Cumilla Bangladesh; ^3^ Department of Clinical Pharmacy and Pharmacology, Faculty of Pharmacy University of Dhaka Dhaka Bangladesh; ^4^ Department of Pharmacy Jashore University of Science and Technology Jessore Bangladesh

**Keywords:** *Baccaurea motleyana*, nutritional, pharmacology, phytochemistry, Rambai, underutilized fruit

## Abstract

Rambai (*Baccaurea motleyana* Müll. Arg.), a member of the Phyllanthaceae family, is one of the underutilized fruits native to Indonesia, Malaya Peninsula, and Thailand. Nowadays, *B. motleyana* is cultivated for its fruits in many parts of Northern Australia, China, and Southeast Asia. The edible part of the fruit is white and contains reddish arillodes that taste sweet to acid‐sweet. The ripe fruit is consumed fresh and can be processed into juice, jams, organic vinegar, and wine. Traditionally, the fruit and its bark are used to treat stomach and eye diseases, respectively. The fruits of *B. motleyana* are a good source of vitamins, minerals, and fibers, and they also contain bioactive compounds such as phenolic acids, flavonoids, carotenoids, and terpenes. This scientific review describes the nutritional composition, phytochemistry, and pharmacology of *B. motleyana*. In addition, most recent information is provided to promote the widespread consumption of *B. motleyana* fruit as well as to create research interest on this interesting species among the scientific community.

## Graphical abstract

*Baccaurea motleyana* Müll. Arg. fruit is a useful but underutilized fruit that has numerous medicinal properties. Here, a short overview of the plant has been given to uncover its therapeutic potential.

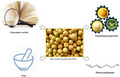

## 1 INTRODUCTION

Fruits contain highly nutritious (vitamins, minerals, fibers) and bioactive substances, which are thought to contribute to beneficial health effects (Li et al., 2016). There are proven links to a lower risk of developing massive serious chronic disorders such as diabetes and cancer with intake of fruits. The World Health Organization (WHO) suggests consuming at least 400 g of fruits every day (Singh et al., 2016). Humans have been cultivating plants primarily as food sources (Khoo et al., 2016). In contrast to commercial fruits, underutilized fruits have gained less interest, probably due to a lack of awareness of their significant benefits (Mirfat et al., 2018). However, the popularity of exotic tropical fruits has risen in recent years in both national and international markets due to awareness about their nutritional worth and link to well‐being (Araújo et al., 2021).

**FIGURE 1:**
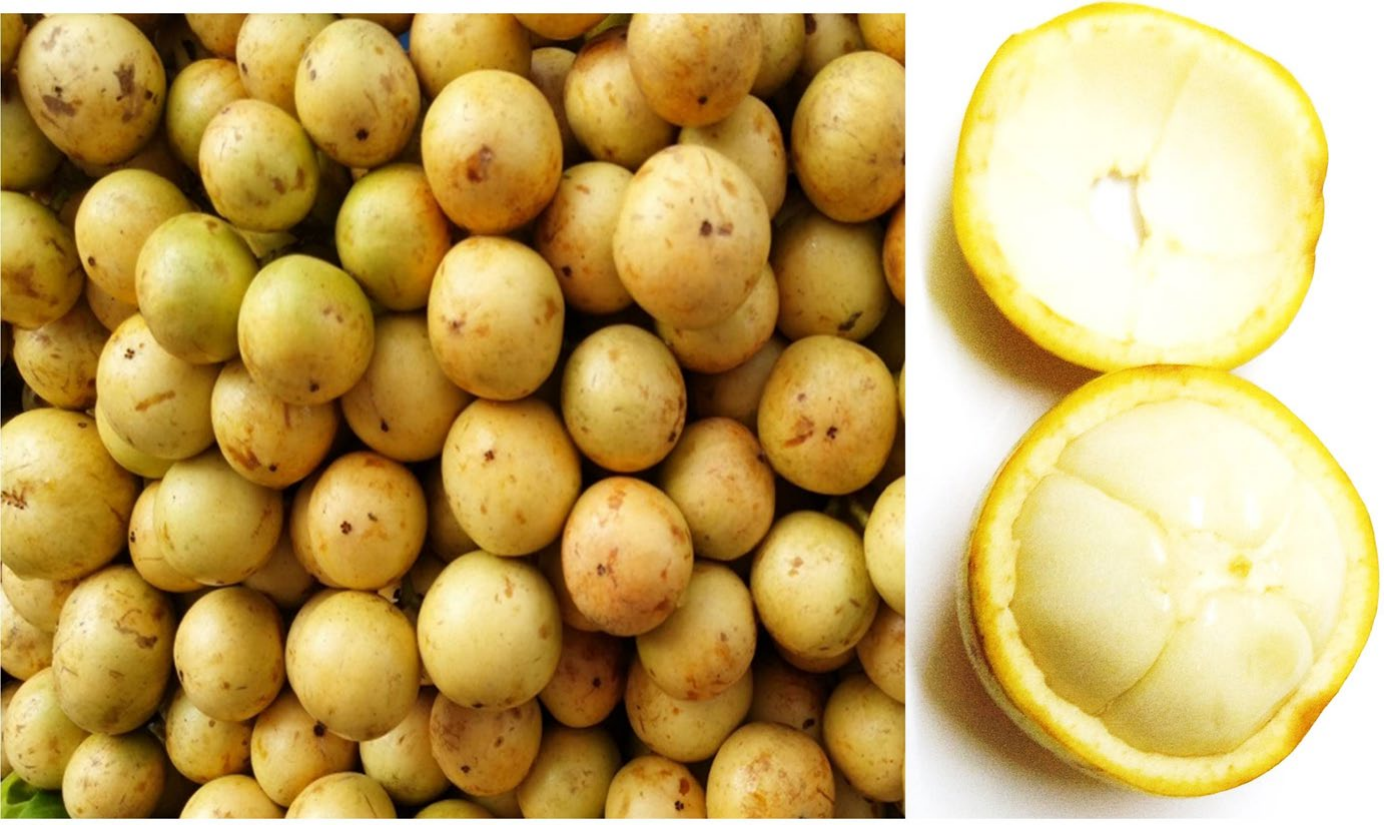
Fruits of *Baccaurea motleyana* (Rambai)

*Baccaurea motleyana* is a member of the Phyllanthaceae family. It is a tropical plant originally found in Kalimantan, Java, Sumatra, Bali, and Peninsular Malaysia (Nurmayani et al., 2021). This tree is called Rambai in English, which is also the local name in Indonesia and Malaysia (Subhadrabandhu, 2001). The Rambai tree is cultivated for its fruit in Southeast Asian countries such as Philippines, Thailand, India, and Bangladesh (Erwin et al., 2020; Nurmayani et al., 2021). The fruit is called as Rambi in the Philippines; mafai‐farang (general), ramai, or lam‐khae (Pattani), and raa‐maa tee‐ku (Narathiwat) in Thailand; Leteku in Assam, India; and as Latkan or “Bubi” in Bangladesh. (Erwin et al., 2020; Khoo et al., 2016; Roy & Khan, 2020; Subhadrabandhu, 2001). Rambai grows in lowland areas, rainforests, plain grassland, and riverbank forest areas in its native environment, and is frequently cultivated in home gardens (Lim, 2012; Nurmayani et al., 2021). It grows at altitudes of 10–750 m on fertile soils, brown mud, silt, or limestone (Lim, 2012). The fruit (Figure 1) is oval‐shaped and is 2.5–4 cm in diameter. The color of the fruit is yellowish‐green when unripe and yellowish‐white when ripe (Lim, 2012; Nurmayani et al., 2021). The fruits are delicious and pleasant; they can be either eaten raw or processed into juice, jams, organic vinegar, and wine (Lim, 2012; Normah, 2003). The fruits are usually grown plentiful and widely available in the marketplace during June to August (Normah, 2003). Rambai fruits are high in vitamins, minerals, fibers, and pharmacologically important substances such as phenolic acids, flavonoids, terpenes, and low amounts of carotenoids (Khoo et al., 2016; Lim, 2012; Susandarini et al., 2021). It was reported that about 46 volatile components have been identified from the essential oil of Rambai fruit using the GC‐MS technique, and (E)‐Hex‐2‐enal was the major component identified from the essential oil (Wong et al., 1994). Furthermore, fruit skin extract was found to have antibacterial activity against *Bacillus cereus*, *Bacillus subtilis*, *Staphylococcus vulgaris*, and *Escherichia coli*, as well as a reduced blood sugar activity that induce hypoxia in mice (Nurmayani et al., 2021; Ramasamy et al., 2011).

As the fruit is less popular compared with other commercially available fruits, limited information is found. In this scientific review, a comprehensive overview on the current nutritional and phytochemical composition of this fruit is provided to create awareness for the commercial growth of this underutilized fruit around the world.

## 2 MATERIALS AND METHODS

All information provided on *B*. *motleyana* was obtained by searching the Web of Science, Google, PubMed, ScienceDirect, Springer, as well as a library search of peer‐reviewed journal papers. The ITIS (2021) database was used to validate the taxonomy of *B. motleyana* (https://www.itis.gov).

## 3 GEOGRAPHICAL DISTRIBUTION

Rambai is native to North Sumatra, Kalimantan, West Java, Borneo, and Peninsular Malaysia (Khoo et al., 2016; Lim, 2012; Nurmayani et al., 2021; Wong et al., 1994). However, the Rambai trees are nowadays cultivated for their fruits in tropical areas elsewhere in Southeast Asia, Northern Australia, and China (Erwin et al., 2020; Ismail et al., 2012; Khoo et al., 2016; Lim, 2012). In Indonesia, the trees are grown in the tropical rain forests of east Kalimantan (Erwin et al., 2020) and also cultivated in different regions, such as Borneo, Sumatra, and Java (Normah, 2003). In Malaysia, the fruit trees are produced from the Malaysian rain forests in Pasoh, Peninsular Malaysia (Wong, 1995). In Bangladesh, the local name of this fruit is Lotkon and Bhubhi (Roy & Khan, 2020) and has been cultivated in different districts such as Narsingdi, Gazipur, Sylhet, and Netrokona.

## 4 TAXONOMY AND MORPHOLOGICAL DESCRIPTION

**FIGURE 2:**
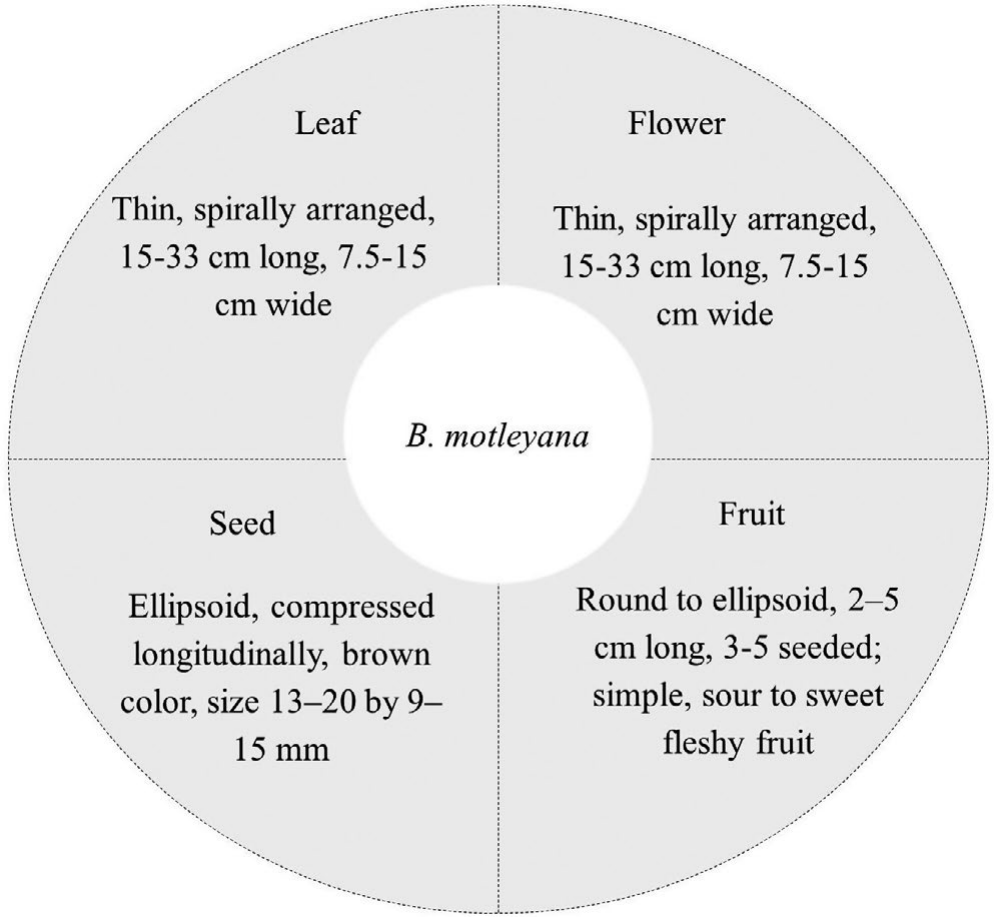
Morphological characterization of *Baccaurea motleyana*

J. Motley, a British engineer, first discovered Rambai plants from the Southeast Borneo Island in 1866. The taxonomy of *B. motleyana* (Table 1) was validated using the ITIS (Integrated Taxonomic Information System) database (ITIS, 2021). The morphological profile of *B. motleyana* is depicted in Figure 2. In brief, the Rambai tree is a densely leafy and large plant that can reach a height of 9–25 m. The tree's trunk can reach 0.4 m in length. This tree has a low, spherical, and bushy crown. The Rambai tree is a strong plant that can thrive in different environments. The leaves are spirally arranged, flat, glossy on the top, and deeper green on the abaxial surface, 15–33 cm long, and 7.5–15 cm wide with 12–16 pairs of lateral veins (plates 2–3). Inflorescence spikes appear on the trunk and branches, seldom on smaller branchlets. The tiny, greenish yellow, male and female apetalous flowers are grown on individual trees. Both types of flowers have yellow sepals and are fragrant. Female racemes are 25–60 cm long, and female blooms are borne in clusters on a regular basis. The pleasant, sweet aroma of the flowers attracts small insects for pollination. Fruit forms in beautiful clusters from the older branches and trunk. The ripe fruits are globose to ellipsoid in shape, 2–5 cm long, and contain three to five seeded indehiscent berries that are greenish yellow to whitish yellow. The edible part of the fruit is the juicy translucent pulp. The taste of the pulp ranges from sweet to sour. The seeds are ellipsoid, compressed longitudinally, and brown, size 13–20 by 9–15 mm. Arillode is opaque, varying from white to purple in color (Ismail et al., 2012; Lim, 2012; Normah, 2003).

**TABLE 1:** Taxonomical classification of *Baccaurea motleyana* (ITIS, 2021)

Kingdom	Plantae
Sub‐kingdom	Viridiplantae
Infra‐kingdom	Streptophyta
Superdivision	Embryophyta
Division	Tracheophyta
Subdivision	Spermatophytina
Class	Magnoliopsida
Superorder	Rosanae
Order	Malpighiales
Family	Phyllanthaceae
Genus	*Baccaurea*
Species	*Baccaurea motleyana* Müll. Arg.

## 5 TRADITIONAL USES

Traditionally, Rambai fruits and peel are used as remedy for stomachache and sore eye diseases, respectively (Erwin et al., 2020; Ismail et al., 2012; Lim, 2012; Ramasamy et al., 2011). Rambai peels are used as ingredients in traditional cosmetic industries of different countries like Malaysia and Indonesia. They are used to cure acne and other skin disorders and are also eaten as a vegetable (Mohamed et al., 1994). Fruits are used to treat bacterial infections (Ramasamy et al., 2011). The crushed fruit rinds are consumed orally to induce sleeping (Ong et al., 2011). The bark has also been included as an ingredient in a variety of preparations and has been given to women as a preventive measure of postpartum hemorrhage (Lim, 2012).

## 6 NUTRITIONAL AND PHYTOCHEMICAL COMPOSITION

### 6.1 Physicochemical and nutritional properties of fruit

The nutritional composition of the fruit depends on various factors such as cultivation methods, geography, climate, plant age, and growth circumstances (Dorais, 2008). Rambai fruits contain water (83.7 g/100 g); categorized as a “fleshy fruit” (Subhadrabandhu, 2001); and are enriched with vital nutrients like vitamins and minerals, as well as physicochemicals like fibers, proteins, carbohydrates, and a low‐fat content (Lim, 2012).

Vitamin C is a vital component for humans, and a deficit causes lowered immunity and increased susceptibility to diseases (Carr & Maggini, 2017). The daily vitamin C requirements are met by eating two to three Rambai fruits. Vitamin C is the major compound compared with other available vitamins, followed by vitamin B_3_. In ripe fruit, potassium is the most abundant mineral compared with other minerals (Leung et al., 1972; Tee et al., 1997). The variation in mineral concentrations in the fruits is influenced by a number of factors, including soil fertility, climate, and maturity stages (Betta et al., 2018).

Carbohydrates are the most abundant component in fruits and have a significant impact on sensory properties, particularly sweetness. Fruits are typically high in fructose and glucose monosaccharides, but their quantity and relative abundance vary by species (Schulz et al., 2020). About 16.1 g carbohydrates was observed in fresh *B*. *motleyana* per 100 g edible portion fruit (Lim, 2012).

Food fibers are an important source of nutrients and have a number of health benefits (Fuller et al., 2016). In addition, food fibers contain a number of bioactive substances that have beneficial effects on the gut flora (Guergoletto et al., 2016). A summary of the nutritional and physicochemical composition of the edible fresh fruit of *B. motleyana* is presented in Table 2.

**TABLE 2:** Physicochemical and nutritional composition of *Baccaurea motleyana* fruit

Physicochemical composition per 100 g							
	Energy	Water	Protein	Fat	Carbohydrate	Ash	Fiber
^a^	64 kcal	83.7%	0.4 g	0.4 g	14.6 g	0.2 g	0.1 g
^b^	65 kcal	79.0%	0.2 g	0.1 g	16.1 g	3 g	0 g

Nutritional composition per 100 g							
Minerals (mg)	^a^	^b^	^c^	Vitamins (mg)	^a^	^b^	^c^
Ca	5	13	5.4	Vitamin B_1_	0.02	–	0.55
P	13	20	19.7	Vitamin B_2_	0.05	–	0.11
Fe	0.2	0.8	–	Vitamin B_3_	0.2	–	–
Na	2	–	–	Vitamin C	6.2	5	11.2
K	111	–	142				
Mg	–	–	11.8				

^a^ Tee et al. (1997).

^b^ Leung et al. (1972).

^c^ Khadijah and Razali (2010).

### 6.2 Phytochemical significance

Numerous internal and external parameters, including species, cultivar, soil, rainfall, intensity of light, maturation stage, and geography, affect the content of the fruits, resulting in variation within and across species (Betta et al., 2018; Taiz & Zeiger, 2009). The phytochemical analysis has discovered about 88 phytochemicals, which are phenolics, carotenoids, and other miscellaneous compounds in the different parts of the Rambai fruit (Susandarini et al., 2021).

#### 6.2.1 Phenolic compounds

Phenolics are nonnutritive compounds that are found in fruits and vegetables and play a significant role in health maintenance (Araújo et al., 2021). They are divided into several classes including phenolic acids, flavonoids, and their hydrolyzed products and derivatives (Rakariyatham et al., 2020). These compounds are known as natural antioxidants, which have therapeutic impacts on health including heart disease and cancer (Mokhtar et al., 2014).

Aside from the dietary and nutritious content, the Rambai fruit also contains several natural antioxidant compounds including phenolics and flavonoids. The phenolic contents of unripe, mature, and ripe Rambai fruits were found to be 97.23 mg/100 g, 63.90 mg/100 g, and 79.57 mg/100 g, respectively (Nurmayani et al., 2021). The total phenolic content (TPC) is one of the most commonly used methods for determining the amount of phenolic antioxidants present in fruits (Khoo et al., 2016). In a previous study, the TPC in Rambai fruit was found to be 1160.14 ± 20.56 (mg GAE/100 g edible portion) (Ikram et al., 2009).

#### 6.2.2 Carotenoids

Carotenoids are a type of phytochemical, which are categorized into carotenes and xanthophylls (Khoo et al., 2016). These are the pigments that give fruits and vegetables their bright colors of yellow, orange, and red. Carotenoid‐rich foods are linked to a lower risk of disease such as cancer, cardiovascular diseases, cataract, and macular degeneration (Rodriguez‐Amaya, 2019).

#### 6.2.3 Volatile organic compounds

The characterization of volatile compounds derived from natural resources is useful since they have therapeutic potential for prevention and treatment of different illnesses (Nuutinen, 2018). Furthermore, they are also utilized in the food, beverage, cosmetics, and perfume industries (Farias et al., 2020).

**FIGURE 3:**
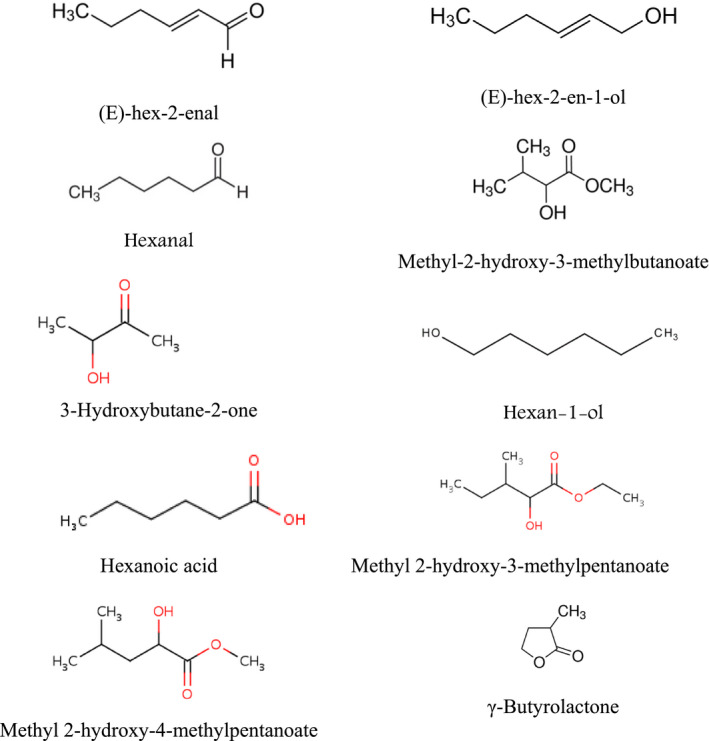
Structure of major volatile compounds identified in *Baccaurea motleyana* fruit

The volatile constituents obtained from the fruits of Rambai were analyzed by capillary GC and GC‐MS (Wong et al., 1994). (E)‐Hex‐2‐enal was the major component of Rambai fruit, which also contained high levels of methyl 2‐hydroxy‐3‐methylbutanoate, methyl 2‐hydroxy‐3‐methylpentanoate and methyl 2‐hydroxy‐4‐methylpentanoatc (Figure 3). Wong et al. (1994) reported that terpenes are the minor components in the essential oil of Rambai. In total, 46 compounds amounting to 97.26% of the sample were identified or partially characterized. Among these were 19 esters, 9 alcohols, 5 aldehydes, 3 ketones, 3 carboxylic acids, 2 phenols, and 5 miscellaneous compounds. The yield of total volatiles was estimated to be 2.0 mg/kg of the fruit pulp. Table 3 lists the volatile compounds identified from Rambai fruit by Wong et al. (1994).

**TABLE 3:** Different volatile compounds of fresh *Baccaurea motleyana* fruit

No.	Compounds	Area (%)	Odor type
1	(E)‐Hex‐2‐enal	55.05	Green type
2	Methyl 2‐hydroxy‐3‐methylbutanoate	8.94	Fruity odor
3	Methyl 2‐hydroxy‐3‐methylpentanoate	7.6	Fruity odor
4	(E)‐Hex‐2‐en‐1‐ol	5.12	Fruity‐green aroma
5	Methyl 2‐hydroxy‐4‐methylpentanoate	4.74	Fruity odor
6	Hexanal	2.76	Fresh green
7	Hexan‐1‐ol	1.44	Herbal
8	Methyl 2‐hydroxy‐3‐methylpentanoate	1.31	Fruity
9	γ‐Butyrolactone	1.2	Creamy
10	Hexanoic acid	1.14	Fatty
11	3‐Hydroxybutane‐2‐one	1.11	Buttery
12	Methyl (E)‐hex‐2‐eonate	0.81	Fatty
13	(Z)‐Hex‐2‐enal	0.8	Green
14	Methyl 3‐(methylthio)propanoate	0.73	Sulfurous
15	2‐Methylbut‐3‐en‐2‐ol	0.55	Herbal
16	Phenol	0.48	Phenolic
17	Pent‐1‐en‐3‐ol	0.42	Green
18	Methyl 2‐methylbutanoate	0.39	Pungent, apple‐like
19	(Z)‐Pent‐2‐en‐1‐ol	0.36	Green
20	Methyl hexanoate	0.33	Fruity
21	Pentan‐3‐one	0.31	Ethereal
22	Pentadecane	0.24	Woody
23	Linalol	0.24	Floral
24	Methyl pyrrole‐2‐carboxylate	0.23	‐
25	(Z)‐Hex‐3‐en‐l‐ol	0.15	Green
26	(Z)‐Hex‐3‐enal	0.12	Green
27	Methyl 2‐hydroxypropanoate	0.12	‐
28	Carvacrol	0.11	Spicy
29	Methyl phenylacetate	0.09	Honey
30	2‐Phenylethanol	0.07	Floral
31	2‐Ethylhexan‐1‐ol	0.06	Fatty
32	2‐Methyltetrahydrofuran‐3‐one	0.03	Bready
33	Methyl furoate	0.03	Caramellic
34	Methyl salicylate	0.03	Minty
35	Methyl (E)‐cinnamate	0.03	Balsamic
36	Methyl (Z)‐hex‐3‐enoate	0.02	Earthy
37	Methyl 3‐hydroxy‐3methylbutanoate	0.02	‐
38	Methyl 2‐hydroxypentanoate	0.02	Truffle
39	Methyl benzoate	0.02	Phenolic
40	Naphthalene	0.02	Pungent
41	(E)‐Hex‐2‐enoic acid	0.02	Fruity
42	Limonene	<0.01	Citrus
43	Linalol oxide, cis‐furanoid	<0.01	Earthy
44	3‐(Methylthio)propanal	<0.01	Vegetable
45	Propanoic acid	<0.01	Acidic
46	Ethyl hexadecanoate	<0.01	Waxy
Total area (%) =		97.26%	

#### 6.2.4 Organic acids

Organic acids including citric, malic, and tartaric acids play an important role in plant growth, maturity, and senescence. Furthermore, fruit juices with a low pH level have a higher amount of organic acids (Karadeniz, 2004). The chemical and physiological features of fruits can be affected by changes in the content of organic acids along the ripening period (Abeles & Takeda, 1990). Diverse organic acids, such as mallic acid, tartaric acid, citric acid, and oxalic acid, have been identified in the ripe fruit of *B. motleyana* (Mokhtar et al., 2014; Susandarini et al., 2021).

## 7 PHARMACOLOGICAL ACTIVITIES

Fruits are widely suggested in our daily diet chart because of their health‐promoting properties (Aprea et al., 2015; Thilakarathna & Vasantha Rupasinghe, 2012). Different scientific studies reported that fruits contain several bioactive compounds including phytochemicals (phenolics, flavonoids, and carotenoids), vitamins (vitamin C, folate, and pro‐vitamin A), minerals (potassium, calcium, and magnesium), and fibers that are known to possess significant therapeutic potential (Kumar et al., 2020; Rangarajan et al., 2021; Venthodika et al., 2021). Experimental evidence from various in vitro and in vivo studies suggest that these bioactive compounds prevent and reduce the risk of chronic diseases such as anti‐inflammatory diseases, hepatoprotective diseases, obesity, diabetes, cardiovascular diseases, and cancer and furthermore have shown positive results in the management of such diseases (Kaur et al., 2019; Khalid et al., 2017).

Scientifically, very few works related to pharmacological activities have been reported to date. However, the antioxidant, antimicrobial, and anticancer potentials have been reported with various parts of *B. motleyana*. The pharmacological activities and works related to this species are tabulated in Table 4.

**TABLE 4:** Pharmacological activities of different parts of *Baccaurea motleyana*

Pharmacological activity	Parts used	Assay/Study type	Extract	Results/Activity	References
Antioxidant capacity	Fruit	β‐carotene bleaching assay	Methanolic	71.17 ± 5.63%	Ikram et al. (2009)
Antimicrobial (Gram‐positive and Gram‐negative bacteria, fungus, and yeast)	Peel	Disc diffusion method	Petroleum ether, chloroform and ethanol	Growth inhibition of *S. aureus*, *B. cereus*, *B. subtilis, E. coli*, *P. aeruginosa*, and *P. vulgaris*	Mohamed et al. (1994)
Anticancer activity	Fruit	MTS assay	Hexane	IC_50_ = 51.0 ± 3.1 μg/ml	Ismail et al. (2012)
		–	Dichloromethane	IC_50_ = 82.4 ± 2.4 μg/ml	
	Peel	–	Hexane	IC_50_ = 43.6 ± 0.3 μg/ml	
		–	Dichloromethane	IC_50_ = 75.0 ± 1.2 μg/ml	

### 7.1 Antioxidant activity

Antioxidants are compounds that scavenge the free radicals and reduce oxidative stress, thereby preserving cellular functions (Rangarajan et al., 2021). The antioxidant capacity of fruit is correlated well with the level of oxygen radical scavengers, such as phenolic compounds (Yuan & Zhao, 2017). Phenolic compounds are good antioxidants found in the flesh of fruits including phenolic acids and flavonoids, whereas flavonoids and lignans are found in the seeds or kernel (Wu & Pike, 2001). The methanolic fruit extract of *B. motleyana* demonstrated considerable antioxidant potential (71.17 ± 5.63%) based on the β‐carotene bleaching assay (Ikram et al., 2009).

### 7.2 Antimicrobial activity

Antimicrobial substances inhibit the growth of microorganisms by hindering the essential pathways, physiological, metabolic, and reproduction activities (Venthodika et al., 2021). Fruits have long been known to be promising natural antimicrobial agents, especially for application in the food industry (Lima et al., 2019). In addition, the essential oils of several fruit extracts are reported to have antimicrobial properties (Fraga, 2002). The fruit extract of *B. motleyana* has also been reported as an active antimicrobial agent. Petroleum ether, chloroform, and ethanol extracts of *B. motleyana* peel possessed antimicrobial activities on several types of bacteria, such as *S. aureus, B. cereus, B. subtilis, E. coli, P. aeruginosa, and P. vulgaris*. Rambai peel showed stronger activity than 50 µg streptomycin and appeared to be a good antimicrobial agent (Khoo et al., 2016; Mohamed et al., 1994; Nurmayani et al., 2021). The volatile compound (E)‐Hex‐2‐enal and methyl‐2‐hydroxy‐3‐methylbutanoate isolated from *B. motleyana* was reported to possess antibacterial activity (Halim et al., 2019).

### 7.3 Anticancer activity

Cancer is the second largest cause of death in the world, accounting for 9.6 million deaths globally in 2018 (Kumar et al., 2020; Twilley et al., 2020). More than 60% of anticancer medicines are natural compounds, with about 25% obtained from plants and another 25% developed from plants (Juárez, 2014; Newman et al., 2002). Furthermore, several types of flavonoids including quercetin, myricetin, kaempferol, and rutin have been shown to suppress cell development and serve as anticancer drugs (Patil & Masand, 2019; Wang et al., 2018). The hexane and dichloromethane extracts of Rambai fruits and peel showed significant activity on human colon cancer cell lines (HT‐29) with IC_50_ values ranging from 43.6 ± 0.3 to 82.4 ± 2.4 μg/ml (Erwin et al., 2020; Ismail et al., 2012).

## 8 INDUSTRIAL AND COMMERCIAL USES

Rambai fruits are used for making jam, juice, and vinegar (Soejarto, 1965). In Indonesian cooking, the pickled fruit is served with curries (Subhadrabandhu, 2001). The bark is rich in tannins and yields a mordant for dyes. A black dammar oozes from the bark (Lim, 2012). Rambai tree timber is of low quality but is used for posts (Ismail et al., 2012; Lim, 2012).

## 9 CONCLUSION AND FUTURE ASPECTS

Southeast Asia, including Indonesia, Malaysia, Thailand, and Bangladesh, comprises nations wealthy in plant biodiversity including different varieties of fruits. Fruit‐rich diets have gained prominence because of the presence of various bioactive components, especially phenolic compounds, terpenes, and other terpenoids. The traditional and translational research have revealed that bioactive components in fruits play an important role in the prevention, management, and treatment of different diseases. Although popular at present in Bangladesh, the Rambai fruit has not received much attention when compared with commercial fruits. One of the reasons may be due to the lack of knowledge of its potential value. Overall, this review summarized the nutritional, phytochemical, and therapeutic potentials of *B. motleyana* for various diseases. The review has provided the current evidence for the presence of different nutritious and bioactive phytochemicals in *B. motleyana*, which could have a role in the prevention and cure of diseases. Further extensive scientific studies are required to evaluate the possible therapeutic applications of *B. motleyana* in the management of various health conditions. The fruit has vast untapped potential that can be exploited by in nutraceutical and pharmaceutical industries, in addition to the development of new functional foods. The information provided in this study can be used to promote the consumption of the underutilized Rambai fruit all over the world.

## CONFLICT OF INTEREST

The authors declare that they have no conflict of interest.

## ETHICAL STATEMENT

Ethical approval regarding animal or human subjects is not applicable as this study does not involve any human or animal testing.

## Data Availability

Data sharing is not applicable to this article as no datasheet was generated or analyzed during the current review.

## References

[fsn32661-bib-0001] Abeles, F. B. , & Takeda, F. (1990). Cellulase activity and ethylene in ripening strawberry and apple fruits. Scientia Horticulturae, 42(4), 269–275. 10.1016/0304-4238(90)90050-o

[fsn32661-bib-0002] Aprea, E. , Biasioli, F. , & Gasperi, F. (2015). Volatile compounds of raspberry fruit: From analytical methods to biological role and sensory impact. Molecules, 20(2), 2445–2474. 10.3390/molecules20022445 25647579 PMC6272157

[fsn32661-bib-0003] Betta, F. D. , Nehring, P. , Seraglio, S. K. T. , Schulz, M. , Valese, A. C. , Daguer, H. , Gonzaga, L. V. , Fett, R. , & Costa, A. C. O. (2018). Phenolic compounds determined by LC‐MS/MS and in vitro antioxidant capacity of Brazilian fruits in two edible ripening stages. Plant Foods for Human Nutrition, 73(4), 302–307. 10.1007/s11130-018-0690-1 30218257

[fsn32661-bib-0004] Carr, A. C. , & Maggini, S. (2017). Vitamin C and immune function. Nutrients, 9(11), 1–25. 10.3390/nu9111211 PMC5707683 29099763

[fsn32661-bib-0005] de Araújo, F. F. , de Paulo Farias, D. , Neri‐Numa, I. A. , Dias‐Audibert, F. L. , Delafiori, J. , de Souza, F. G. , Catharino, R. R. , do Sacramento, C. K. , & Pastore, G. M. (2021). Chemical characterization of *Eugenia stipitata*: A native fruit from the Amazon rich in nutrients and source of bioactive compounds. Food Research International (Ottawa, Ont.), 139, 109904. 10.1016/j.foodres.2020.109904 33509473

[fsn32661-bib-0006] de Paulo Farias, D. , Neri‐Numa, I. A. , de Araújo, F. F. , & Pastore, G. M. (2020). A critical review of some fruit trees from the Myrtaceae family as promising sources for food applications with functional claims. Food Chemistry, 306, 125630. 10.1016/j.foodchem.2019.125630 31593892

[fsn32661-bib-0007] Dorais, M. (2008). Improving the health‐promoting properties of fruit and vegetable products. In F. A. Tomás‐Barberán & M. I. Gil , Agronomy and the nutritional quality of fruit (1st ed., pp. 346–391). 10.1533/9781845694289.4.346

[fsn32661-bib-0008] Erwin , Tonapa, Z. G. , & Alimuddin (2020). Toxicity assay of *Baccaurea motleyana* mull. arg. wood extracts (rambai) and chemical compounds evaluation for the most active fraction. Research Journal of Pharmacy and Technology, 13(11), 5215–5218. 10.5958/0974-360X.2020.00912.9

[fsn32661-bib-0009] Fraga, B. M. (2002). Natural sesquiterpenoids. Natural Product Reports, 19(5), 650–672. 12430727 10.1039/b108977n

[fsn32661-bib-0010] Fuller, S. , Beck, E. , Salman, H. , & Tapsell, L. (2016). New horizons for the study of dietary fiber and health: A review. Plant Foods for Human Nutrition, 71(1), 1–12. 10.1007/s11130-016-0529-6 26847187

[fsn32661-bib-0011] Guergoletto, K. B. , Costabile, A. , Flores, G. , Garcia, S. , & Gibson, G. R. (2016). In vitro fermentation of juçara pulp (*Euterpe edulis*) by human colonic microbiota. Food Chemistry, 196, 251–258. 10.1016/j.foodchem.2015.09.048 26593490

[fsn32661-bib-0012] Halim, H. R. , Hapsari, D. P. , Junaedi, A. , Ritonga, A. W. , Natawijaya, A. , Poerwanto, R. , Sobir , Widodo, W. D. , & Matra, D. D. (2019). Metabolomics dataset of underutilized Indonesian fruits; rambai (*Baccaurea motleyana*), nangkadak (*Artocarpus nangkadak*), rambutan (*Nephelium lappaceum*) and Sidempuan salak (*Salacca sumatrana*) using GCMS and LCMS. Data in Brief, 23, 103706. 10.1016/j.dib.2019.103706 31372379 PMC6660448

[fsn32661-bib-0013] Ikram, E. H. K. , Eng, K. H. , Jalil, A. M. M. , Ismail, A. , Idris, S. , Azlan, A. , Nazri, H. S. M. , Diton, N. A. M. , & Mokhtar, R. A. M. (2009). Antioxidant capacity and total phenolic content of Malaysian underutilized fruits. Journal of Food Composition and Analysis, 22(5), 388–393. 10.1016/j.jfca.2009.04.001

[fsn32661-bib-0014] Ismail, M. , Bagalkotkar, G. , Iqbal, S. , & Adamu, H. A. (2012). Anticancer properties and phenolic contents of sequentially prepared extracts from different parts of selected medicinal plants indigenous to Malaysia. Molecules, 17(5), 5745–5756. 10.3390/molecules17055745 22628046 PMC6268718

[fsn32661-bib-0015] ITIS (2021). The integrated taxonomic information system (ITIS). Retrieved from https://www.itis.gov/servlet/SingleRpt/SingleRpt?search_topic=TSN&search_value=506426#null

[fsn32661-bib-0016] Juárez, P. (2014). Plant‐derived anticancer agents: A promising treatment for bone metastasis. BoneKEy Reports, 3, 599. 10.1038/bonekey.2014.94 28243436 PMC5307970

[fsn32661-bib-0017] Karadeniz, F. (2004). Main organic acid distribution of authentic citrus juices in Turkey. Turkish Journal of Agriculture and Forestry, 28, 267–271.

[fsn32661-bib-0018] Kaur, K. , Chhikara, N. , Sharma, P. , Garg, M. K. , & Panghal, A. (2019). Coconut meal: Nutraceutical importance and food industry application. Foods and Raw Materials, 7(2), 419–427. 10.21603/2308-4057-2019-2-419-427

[fsn32661-bib-0019] Khadijah, A. , & Razali, A. R. (2010). Distribution and diversity of *Baccaurea motleyana* in Malaysia. Poster session presentation at the 2nd National Conference on Agrobidoversity, Conservation and Sustainable Utilization (NAC‐2) at Tawau, Sabah, Malaysia. 10.13140/RG.2.2.20209.58721

[fsn32661-bib-0020] Khalid, S. , Khalid, N. , Khan, R. S. , Ahmed, H. , & Ahmad, A. (2017). A review on chemistry and pharmacology of Ajwa date fruit and pit. Trends in Food Science & Technology, 63, 60–69. 10.1016/j.tifs.2017.02.009

[fsn32661-bib-0021] Khoo, H. E. , Azlan, A. , Kong, K. W. , & Ismail, A. (2016). Phytochemicals and medicinal properties of indigenous tropical fruits with potential for commercial development. Evidence‐Based Complementary and Alternative Medicine, 2016, 1–20. 10.1155/2016/7591951 PMC4906201 27340420

[fsn32661-bib-0022] Kumar, H. , Bhardwaj, K. , Dhanjal, D. S. , Nepovimova, E. , Șen, F. , Regassa, H. , Singh, R. , Verma, R. , Kumar, V. , Kumar, D. , Bhatia, S. K. , & Kuča, K. (2020). Fruit extract mediated green synthesis of metallic nanoparticles: A new avenue in pomology applications. International Journal of Molecular Sciences, 21(22), 8458. 10.3390/ijms21228458 PMC7697565 33187086

[fsn32661-bib-0023] Leung, W.‐T.‐W. , Butrum, R. R. , Huang, C. F. , Narayana, R. M. , & Polacchi, W. (1972). Food composition table for use in East Asia (p. 347). FAO.

[fsn32661-bib-0024] Li, Y. , Zhang, J. J. , Xu, D. P. , Zhou, T. , Zhou, Y. , Li, S. , & Li, H. B. (2016). Bioactivities and health benefits of wild fruits. International Journal of Molecular Sciences, 17(8), 1258. 10.3390/ijms17081258 PMC5000656 27527154

[fsn32661-bib-0025] Lim, T. K. (2012). *Baccaurea motleyana*. Edible medicinal and non‐medicinal plants (pp. 239–242). Springer. 10.1007/978-94-007-4053-2_33

[fsn32661-bib-0026] Lima, M. C. , Paiva de Sousa, C. , Fernandez‐Prada, C. , Harel, J. , Dubreuil, J. D. , & de Souza, E. L. (2019). A review of the current evidence of fruit phenolic compounds as potential antimicrobials against pathogenic bacteria. Microbial Pathogenesis, 130, 259–270. 10.1016/j.micpath.2019.03.025 30917922

[fsn32661-bib-0027] Mirfat, A. H. S. , Amin, I. , Kartinee, K. N. , Muhajir, H. , & Shukri, M. A. M. (2018). Underutilised fruits: A review of phytochemistry and biological properties. Journal of Food Bioactives, 1(1), 2–30. 10.31665/JFB.2018.1124

[fsn32661-bib-0028] Mohamed, S. , Hassan, Z. , & Hamid, N. A. (1994). Antimicrobial activity of some tropical fruit wastes (Guava, Starfruit, Banana, Papaya, Passionfruit, Langsat, Duku, Rambutan and Rambai). Pertanika Journal of Tropical Agricultural Science, 17(3). 219–227. ISSN 0126‐6128

[fsn32661-bib-0029] Mokhtar, S. I. , Leong, P. C. , Ven, L. E. , & Nur Ain, A. A. (2014). Total phenolic contents, antioxidant activities and organic acids composition of three selected fruit extracts at different maturity stages. Journal of Tropical Resources and Sustainable Science, 2, 40–46. ISSN 2289‐3946

[fsn32661-bib-0030] Newman, D. J. , Cragg, G. M. , Holbeck, S. , & Sausville, E. A. (2002). Natural products and derivatives as lead to cell cycle pathway targets in cancer chemotherapy. Current Cancer Drug Targets, 2(4), 279–308. 10.2174/1568009023333791 12470208

[fsn32661-bib-0031] Normah, M. N. (2003). Fruits of tropical climates | lesser‐known fruits of Asia. Encyclopedia of Food Sciences and Nutrition, 2816–2820. 10.1016/b0-12-227055-x/01403-6

[fsn32661-bib-0032] Nurmayani, S. , Widodo, W. D. , & Matra, D. D. (2021). Characterization of rambai (*Baccaurea motleyana*) genes putatively involved in sugar metabolism. IOP Conference Series: Earth and Environmental Science, 694, 012067. 10.1088/1755-1315/694/1/012067

[fsn32661-bib-0033] Nuutinen, T. (2018). Medicinal properties of terpenes found in *Cannabis sativa* and *Humulus lupulus* . European Journal of Medicinal Chemistry, 157, 198–228. 10.1016/j.ejmech.2018.07.076 30096653

[fsn32661-bib-0034] Ong, H. C. , Ahmad, N. , & Milow, P. (2011). Traditional medicinal plants used by the temuan villagers in Kampung Tering, Negeri Sembilan, Malaysia. Studies on Ethno‐Medicine, 5(3), 169–173. 10.1080/09735070.2011.11886406

[fsn32661-bib-0035] Patil, V. M. , & Masand, N. (2019). Anticancer potential of flavonoids: Chemistry, biological activities, and future perspectives. In A. Rahman (Ed.), Studies in natural products chemistry (1st ed., pp. 401–430). ISBN 1572‐5995.

[fsn32661-bib-0036] Rakariyatham, K. , Zhou, D. , Rakariyatham, N. , & Shahidi, F. (2020). Sapindaceae (*Dimocarpus longan* and *Nephelium lappaceum*) seed and peel by‐products: Potential sources for phenolic compounds and use as functional ingredients in food and health applications. Journal of Functional Foods, 67, 103846. 10.1016/j.jff.2020.103846

[fsn32661-bib-0037] Ramasamy, S. , Wahab, N. A. , Abidin, N. Z. , & Sugumaran, M. (2011). Cytotoxicity evaluation of five selected Malaysian Phyllanthaceae species on various human cancer cell lines. Journal of Medicinal Plant Research, 5, 2267–2273. 10.5897/JMPR.9000432

[fsn32661-bib-0038] Rangarajan, H. , Elumalai, A. , & Chidanand, D. V. (2021). Traditional fruits of South India: Bioactive components and their potential health implications in chronic diseases. Journal of Food Biochemistry, 45(3), e13266. 10.1111/jfbc.13266 32529677

[fsn32661-bib-0039] Rodriguez‐Amaya, D. B. (2019). Update on natural food pigments ‐ A mini‐review on carotenoids, anthocyanins, and betalains. Food Research International, 124, 200–205. 10.1016/j.foodres.2018.05.028 31466641

[fsn32661-bib-0040] Roy, G. K. , & Khan, S. A. (2020). Preliminary taxonomic study on homestead flora of four districts of Bangladesh: Magnoliopsida. Bangladesh Journal of Plant Taxonomy, 27(1), 37–65. 10.3329/bjpt.v27i1.47567

[fsn32661-bib-0041] Schulz, M. , Seraglio, S. K. T. , Brugnerotto, P. , Gonzaga, L. V. , Costa, A. C. O. , & Fett, R. (2020). Composition and potential health effects of dark‐colored underutilized Brazilian fruits – A review. Food Research International, 137, 109744. 10.1016/j.foodres.2020.109744 33233309

[fsn32661-bib-0042] Singh, B. , Singh, J. P. , Kaur, A. , & Singh, N. (2016). Bioactive compounds in banana and their associated health benefits ‐ A review. Food Chemistry, 206, 1–11. 10.1016/j.foodchem.2016.03.033 27041291

[fsn32661-bib-0043] Soejarto, D. D. (1965). Baccaurea and its uses. Botanical Museum Leaflets, Harvard University, 21(3), 65–104. Retrieved from http://www.jstor.org/stable/41762245

[fsn32661-bib-0044] Subhadrabandhu, S. (2001). Under‐utilized tropical fruits of Thailand (70 p). FAO Regional Office for Asia and the Pacific.

[fsn32661-bib-0045] Susandarini, R. , Khasanah, U. , & Rosalia, N. (2021). Ethnobotanical study of plants used as food and for maternal health care by the Malays communities in Kampar Kiri Hulu, Riau. Indonesia. Biodiversitas Journal of Biological Diversity, 22(6), 3111–3120. 10.13057/biodiv/d220613

[fsn32661-bib-0046] Taiz, L. , & Zeiger, E. (2009). Fisiologia Vegetal, (4th ed.). Artmed.

[fsn32661-bib-0047] Tee, E. S. , Noor, M. I. , Azudin, M. N. , & Idris, K. (1997). Nutrient composition of Malaysian foods. In Institute for Medical Research (4th ed., p. 299). Ministry of Health. ISBN 967‐99909‐8‐2

[fsn32661-bib-0048] Thilakarathna, S. H. , Vasantha Rupasinghe, H. P. (2012). Antiatherosclerotic effects of fruit bioactive compounds: A review of current scientific evidence. Canadian Journal of Plant Science, 92(3), 407–419. 10.4141/cjps2011-090

[fsn32661-bib-0049] Twilley, D. , Rademan, S. , & Lall, N. (2020). A review on traditionally used South African medicinal plants, their secondary metabolites and their potential development into anticancer agents. Journal of Ethnopharmacology, 261, 113101. 10.1016/j.jep.2020.113101 32562876

[fsn32661-bib-0050] Venthodika, A. , Chhikara, N. , Mann, S. , Garg, M. K. , Sofi, S. A. , & Panghal, A. (2021). Bioactive compounds of *Aegle marmelos* L., medicinal values, and its food applications: A critical review. Phytotherapy Research, 35(4), 1887–1907. 10.1002/ptr.6934 33159390

[fsn32661-bib-0051] Wang, T. Y. , Li, Q. , & Bi, K. S. (2018). Bioactive flavonoids in medicinal plants: Structure, activity and biological fate. Asian Journal of Pharmaceutical Sciences, 13(1), 12–23. 10.1016/j.ajps.2017.08.004 32104374 PMC7032191

[fsn32661-bib-0052] Wong, K. C. (1995). Collection and evaluation of under‐utilized tropical and subtropical fruit tree genetic resources in Malaysia. In JIRCAS International Symposium Series (Vol. 3, pp. 27–38).

[fsn32661-bib-0053] Wong, K. C. , Wong, S. W. , Siew, S. S. , & Tie, D. Y. (1994). Volatile constituents of the fruits *Oflansium domesticum* correa (Duku and Langsat) and *Baccaurea motleyana* (Muell. Arg.) Muell. Arg. (Rambai). Flavour and Fragrance Journal, 9(6), 319–324. 10.1002/ffj.2730090608

[fsn32661-bib-0054] Wu, A. H. , & Pike, M. C. (2001). Phytoestrogen content in foods and their role in cancer. In Handbook of antioxidants, revised and expanded. Marcel Dekker.

[fsn32661-bib-0055] Yuan, Q. , & Zhao, L. (2017). The Mulberry (*Morus alba* L.) fruit—a review of characteristic components and health benefits. Journal of Agricultural and Food Chemistry, 65(48), 10383–10394. 10.1021/acs.jafc.7b03614 29129054

